# Artificial intelligence in ACL injury prediction and prevention: a systematic review

**DOI:** 10.1186/s13018-026-06825-0

**Published:** 2026-04-07

**Authors:** Mohammad Reza Molavi, Mohamad Mottaghitalab, Parsa Samaei, Hamed Zarei

**Affiliations:** 1 Sports Biomechanics Department, College of Physical Education and Sport Sciences, Faculty of Physical Education and Sport Sciences, University of Guilan, Kilometers 10 Rasht-Ghazvin Road, Rasht, 4199613776 Iran; 2 Department of Sport Injuries and Corrective Exercises, Faculty of Physical Education and Sport Sciences, University of Tehran, Tehran, 4167977787 Iran

**Keywords:** Anterior cruciate ligament, Artificial intelligence, Injury prediction, Injury prevention

## Abstract

**Background:**

Anterior cruciate ligament (ACL) injuries are prevalent in sports, with significant physical, economic, and long-term health impacts. Artificial intelligence (AI) offers promising solutions for predicting and preventing ACL injuries through advanced data analysis. This systematic review evaluates AI applications in ACL injury prediction and prevention, focusing on techniques, performance metrics, sports contexts, and intervention effectiveness.

**Methods:**

Following PRISMA 2020 guidelines, we searched PubMed, Scopus, Google Scholar, and Web of Science from inception to November 1, 2025 for peer-reviewed studies in English using AI for ACL injury prediction or prevention. Studies were excluded if they focused on other injuries or were non-original research. Two reviewers independently screened articles, extracted data (e.g., AI techniques, sample size, outcomes), and assessed methodological quality using the PROBAST + AI. Narrative synthesis was conducted due to methodological heterogeneity**.**

**Results:**

Seven studies, published between 2019 and 2024, were included, involving 5–880 participants (age range 13–22.8 years) across sports like basketball, handball, and soccer. AI techniques included machine learning (e.g., support vector machines, random forest) and deep learning (e.g., convolutional neural networks), with applications in risk prediction and biomechanical assessment. Predictive models achieved accuracies of 79.5–96% and AUCs of 0.63–0.85, while prevention-focused studies reported high validity (e.g., R^2^: 0.9947–0.9992). Input data ranged from biomechanical parameters to video-based knee angles. PROBAST + AI demonstrated low ROB, indicating robust methodological quality for development.

**Conclusion:**

AI demonstrates significant potential in predicting ACL injury risk and informing prevention strategies through biomechanical and kinematic analyses. However, small sample sizes, heterogeneous methodologies, and practical barriers (e.g., equipment costs) limit clinical adoption. Future research should focus on larger, diverse cohorts and standardized protocols to enhance generalizability and implementation.

**Registry number:**

CRD420251230914.

**Supplementary Information:**

The online version contains supplementary material available at 10.1186/s13018-026-06825-0.

## Introduction

Anterior cruciate ligament (ACL) injuries rank among the most prevalent and debilitating injuries in sports, particularly in high-impact disciplines such as soccer, basketball, and football [1–3]. These injuries often necessitate surgical intervention, such as ACL reconstruction, followed by a recovery period typically spanning 6–12 months [4–6]. Despite successful surgical outcomes, many athletes struggle to return to their pre-injury performance levels, with approximately 74.5% resuming competitive sport [7, 8]. Moreover, ACL injuries are associated with a significant risk of secondary injuries, with rates of re-injury or contralateral ACL tears reported as high as 20% in some populations [9, 10]. The long-term consequences are equally concerning, with up to 87% of individuals developing post-traumatic osteoarthritis (PTOA) within years of the initial injury, leading to chronic pain and reduced quality of life [11–13]. Additionally, ACL injuries impose a substantial economic burden, encompassing direct medical costs and indirect costs such as lost productivity and prolonged rehabilitation [14, 15]. Despite advances in rehabilitation protocols and the use of various functional assessment tools, the incidence of ACL injuries remains high [16], underscoring the urgent need for more effective prediction and prevention strategies.

Artificial intelligence (AI) has emerged as a transformative tool in healthcare, offering innovative solutions for diagnostics, treatment planning, and outcome prediction [17]. In orthopedics, AI applications include image analysis for fracture detection, predictive modeling for surgical outcomes, and real-time monitoring of patient recovery [18–20]. Specifically, in the realm of ACL injuries, AI has shown promise in enhancing both prediction and prevention efforts. Machine learning (ML) models, for instance, have been developed to predict ACL injury risk by analyzing biomechanical data from motion capture and wearable sensors, achieving accuracies exceeding 90% in some studies [21]. Similarly, deep learning algorithms have demonstrated high diagnostic accuracy, comparable to that of radiologists, in identifying ACL tears from MRI scans [22, 23]. Beyond ACL-specific applications, AI has been successfully employed in other sports to predict injury risks by analyzing player workload and movement patterns, such as in soccer and basketball [24]. These advancements suggest that AI could revolutionize sports medicine by enabling data-driven, personalized approaches to injury prevention.

The integration of AI into sports medicine holds significant potential for transforming ACL injury prevention strategies [25]. By leveraging large datasets from wearable technology, motion capture systems, and clinical records, AI can identify subtle patterns and risk factors that traditional methods may overlook [26]. This capability facilitates the development of personalized training and rehabilitation programs tailored to individual athletes’ biomechanical profiles, potentially reducing ACL injury rates [27]. Furthermore, real-time monitoring systems powered by deep learning algorithms can provide immediate feedback to coaches and medical staff, enabling timely interventions to mitigate high-risk situations before injuries occur [28]. Such advancements could enhance athlete safety and prolong careers, addressing both the physical and economic burdens of ACL injuries.

Injury prediction research has evolved from traditional statistical approaches, such as logistic regression and decision trees, which rely on linear or simple nonlinear assumptions to model relationships between risk factors (e.g., training load, prior injury history, and biomechanical variables) and injury outcomes [29, 30]. These methods offer high interpretability and serve as robust benchmarks but often struggle with complex, high-dimensional, and nonlinear interactions inherent in sports injury etiology [31, 32]. In contrast, modern ML and AI techniques including ensemble methods like random forest and extreme gradient boosting (XGBoost), Support Vector Machines, as well as deep learning architectures such as neural networks (e.g., recurrent or convolutional variants for time-series data) enable more sophisticated pattern recognition, better handling of multifaceted data sources (e.g., wearable sensors, workload metrics), and improved predictive performance in many contexts [29]. Recent systematic reviews highlight that tree-based ensemble models, particularly XGBoost and random forest, frequently outperform traditional approaches in sports injury risk prediction, though challenges remain in model interpretability, generalizability, and clinical translation [33].

However, the current literature on AI applications for ACL injury prediction and prevention is fragmented, with studies employing diverse methodologies, sample sizes, and focus areas. For example, a 2020 systematic review identified only two studies specifically addressing AI in ACL injury prediction, indicating a scarcity of comprehensive analyses [27]. Moreover, inconsistencies in study design, such as variations in AI techniques and performance metrics, limit the ability to draw robust conclusions about AI’s clinical utility [34]. The rapid evolution of AI technologies since 2020, coupled with the increasing incidence of ACL injuries, necessitates an updated and comprehensive synthesis of the evidence. This systematic review aims to address these gaps by evaluating the impact of AI on the prediction and prevention of ACL injuries. Following the PRISMA 2020 guidelines, we will systematically search and analyze the literature to address the following research questions: (1) What AI techniques (e.g., ML algorithms such as random forests, gradient boosting machines like XGBoost, support vector machines, neural networks, or deep learning models including convolutional and recurrent architectures) have been employed for predicting ACL injury risk? (2) What are the reported performance metrics (e.g., AUC, accuracy, sensitivity, specificity, F1-score, calibration plots) of these AI models, and how do they vary across studies, populations, or validation approaches? (3) In which sports contexts, populations (e.g., elite vs. recreational athletes, female athletes, youth), or settings have these AI applications been investigated? (4) What evidence supports the effectiveness of AI-based interventions or tools (e.g., real-time monitoring via wearables, markerless motion analysis, or predictive decision support) in preventing primary or secondary ACL injuries, including outcomes like injury rate reduction or clinical implementation feasibility? By addressing these questions, this review seeks to provide a clear understanding of the current state of AI applications in this field, identify best practices, and highlight areas for future research and clinical implementation. As the use of AI in medical and therapeutic applications continues to grow, it appears that AI could play a pivotal role in predicting and preventing ACL injuries, offering a promising avenue to reduce their incidence and impact.

## Methods

This systematic review was conducted in accordance with the preferred reporting items for systematic reviews and meta-analyses (PRISMA) guidelines to ensure transparency and rigor in reporting [35]. The objective of this review was to evaluate the application of AL, including ML and deep learning, in the prediction and prevention of ACL injuries in athletes. The research questions focused on identifying the AI techniques used, the types of sports examined, and the performance metrics of predictive models. The review protocol has been registered in PROSPERO with the identification number CRD420251230914.

### Eligibility criteria

Studies were included if they were original research articles published in peer-reviewed journals, written in English, and specifically utilized AI techniques for the prediction or prevention of ACL injuries. Studies focusing on other injuries, review articles, conference abstracts, or theses were excluded.

### Search strategy

A comprehensive search was conducted in the PubMed, Scopus, and Web of Science databases, covering the period from inception to November 1, 2025. The search strategy combined the following keywords: (“artificial intelligence” OR “machine learning” OR “deep learning”) AND (“ACL injury” OR “anterior cruciate ligament” OR “ACL”) AND (“prediction” OR “prevention” OR “risk assessment”). The “AND” operator was used to connect the three groups of keywords, while the “OR” operator was employed within each individual keyword group. Additionally, Google Scholar was utilized to broaden our search, incorporating articles from various databases. Following the selection process, we examined the references of the included studies to identify any potentially overlooked citations.

### Study selection

The inclusion criteria were defined as follows: (1) Population: Athletes (professional, collegiate, recreational, or youth) at risk of or with history of ACL injury, or individuals undergoing ACL-related imaging, biomechanical assessment, or monitoring where AI was applied; (2) Intervention: Application of AI techniques (including ML algorithms such as random forests, gradient boosting/XGBoost, support vector machines, neural networks, deep learning models like convolutional neural networks for imaging, or hybrid approaches) for ACL injury prediction (e.g., risk stratification, forecasting based on biomechanical, workload, or clinical data) or prevention (e.g., AI-driven tools for real-time feedback, motion analysis, personalized training adjustments, or wearable-based risk mitigation); (3) Comparator: Not strictly required due to the field's emphasis on model development, validation, and diagnostic/predictive performance; where applicable, comparisons included traditional statistical methods, clinician performance, non-AI approaches, or no comparator (e.g., in pure modeling or diagnostic accuracy studies); (4) Outcomes: Clinically relevant metrics for prediction (e.g., area under the receiver operating characteristic curve [AUC], sensitivity, specificity, accuracy, F1-score, calibration, positive/negative predictive values) and/or prevention (e.g., evidence of reduced injury incidence/rates, improved risk mitigation, biomechanical corrections, or feasibility/implementation outcomes in preventive contexts); secondary outcomes included model interpretability, generalizability, or comparison to human experts; (5) Study Designs: Original peer-reviewed research articles (including prospective/retrospective cohort studies, cross-sectional validation studies, diagnostic accuracy studies, modeling/development studies, and randomized or non-randomized interventional trials where AI was tested for prevention); exclusion of reviews, conference abstracts, theses, non-English publications, or studies without direct AI application to ACL-specific prediction or prevention; (6) Peer-reviewed articles published in English. Duplicate articles were removed using reference management software (EndNote). Two independent reviewers screened the titles and abstracts to identify potentially relevant studies. Full-text articles were then assessed for eligibility based on the inclusion and exclusion criteria. Discrepancies between reviewers were resolved through discussion or consultation with two additional reviewers. The study selection process is presented in a PRISMA flow diagram, summarizing the number of records identified, screened, and included Data were extracted independently by two reviewers using a standardized form. The extracted data included the lead author and year of publication, input features, sample size, gender, age range, athlete type, study goal, type of AI system, primary outcome measures (e.g., accuracy, AUC, F1-score), and key results (Table 1).

**Table 1:** Summary of included studies about AI in the prediction and prevention of ACL injury

References	Input feature	Sample size	Gender	Age range (years)	Athlete type	Goal	Type of system/AI	Primary outcome	Result
Taborri et al. [36]	Leg stability, leg mobility, load absorption capability	39	Female basketball player	13 ± 1	Basketball players	Predict acl injury risk	Machine-learning (Linear SVM)	Accuracy: 96%, F1-score: 95%, Goodness index: 0.08	A machine-learning model using inertial sensors and optoelectronic bars accurately identifies female basketball players at high ACL injury risk, with ellipse area, RMSxy, and θymax as key predictors
Benjaminse et al. [37]	Full-body joint kinematics (mean, peak, and at peak KAM	32	Female	14.8 ± 1.0	Talented female football (soccer) players	Predict knee joint loading during agility tasks	Machine-learning (Fine Gaussian SVM)	Classification accuracy: 79.5% (mean kinematics), AUC: 0.85	The study demonstrates that machine-learning models can classify high and low knee abduction moments (KAMs) during agility tasks with good accuracy. This approach can be used to identify players at higher risk of ACL injury in an ecologically valid environment
Ohnishi et al. [38]	Knee flexion and valgus angles estimated using stretch sensors	5	Mixed (3 males, 2 females)	22.8 ± 1.0	Healthy Participants	Predict ACL injury risk by estimating knee joint angles	Machine-learning (Random Forest Regressor)	Knee flexion angle MAE: 8.73°, Knee valgus angle MAE: 0.81°	The study proposes a knee supporter with stretch sensors to estimate knee flexion and valgus angles, which can help identify athletes at higher risk of ACL injury
Xu et al. [39]	Ankle initial contact angle (AIC), Ankle range of motion (AROM)	56	Male	22.56 ± 5.13	Healthy Participants	Predict ACL force during single-leg landing (SL)	Machine-learning (Sparrow Search Algorithm (SSA) optimized Extreme Learning Machine (ELM) and Long Short-Term Memory (LSTM))	PAF prediction: R2 = 0.9992, MSE = 0.0023, RMSE = 0.0474; ACL force waveform prediction: R2 = 0.9947, MSE = 0.0076, RMSE = 0.0873	The study demonstrates that a machine-learning model optimized with SSA can accurately predict ACL force during single-leg landing, identifying risk factors for ACL injury
Jauhiainen et al. [40]	Anthropometric, clinical, neuromuscular, biomechanical, and genetic data	880	Female	21 ± 4	Elite Handball and Soccer Players	Predict ACL injury risk	Machine-learning (Random Forest, L2-regularized Logistic Regression, Support Vector Machines)	Mean AUC-ROC: 0.63 (Linear SVM)	The study demonstrates that while statistically significant predictive ability exists, the predictive ability remains too low for clinical risk assessment
Johnson et al. [41]	Marker-based motion capture data	458,372 trials (from 2001 to 2017)	Mixed (male and female)	21 ± 4	Young healthy athletic population	Predict 3D knee joint moments (KJM) for ACL injury risk assessment	Deep Learning (Convolutional Neural Network—CaffeNet)	Mean correlation to source modeling: 0.8895 (sidestepping)	The study demonstrates the feasibility of using deep learning to predict KJM directly from motion capture data, providing an early warning system for ACL injury risk
Asaeda et al. [42]	Knee joint angle data from video frames using MediaPipe Pose	15	Male	19.9 ± 1.0	Healthy Adult Men	Calculate knee valgus angle during single-leg drop landing	Machine Learning (MediaPipe Pose)	Concurrent validity with VICON: Change from initial contact (IC) showed good reliability and validity	The study demonstrates that MediaPipe Pose can reliably and validly estimate knee valgus angles, with better results when normalized by the angle at initial contact (IC)

### Quality assessment

This review evaluates the methodological quality and applicability of seven studies that developed AI and ML models to predict the risk of ACL injuries, focusing on biomechanical parameters such as knee abduction moment (KAM) and valgus angle. The assessment utilized the PROBAST + AI tool, an extension of the original PROBAST framework designed to address AI-specific issues, including data preprocessing, model development, and generalizability [43]. PROBAST + AI assesses risk of bias (ROB) and applicability across four domains: Participants, Predictors, Outcome, and Analysis, with ratings assigned as low, high, or unclear. An overall ROB is classified as low if all domains are rated low, high if any domain is rated high, and unclear otherwise. Applicability is evaluated in relation to the review question framed by the PICOTS criteria, which includes the population of athletes at risk for ACL injuries, the intervention of AI/ML models, no comparator, the outcome of ACL injury risk prediction, timing during sports or training, and settings in lab, field, or clinical environments. Notably, all studies were development-focused and lacked external validation, employing various ML techniques such as regression, classification, and deep learning on biomechanical data. Reviews were conducted independently by two reviewers, with discrepancies resolved through consensus. Key challenges identified included small sample sizes, the restriction of lab-based data limiting field applicability, risks of overfitting in ML models, and inadequate management of AI-specific biases, such as data leakage and hyperparameter tuning.

### Data synthesis

Heterogeneity among the included studies was identified through qualitative assessment during data extraction and quality appraisal, focusing on substantial differences in clinical (e.g., populations, sports contexts, risk factors), methodological (e.g., study designs, validation approaches, sample sizes), and statistical aspects (e.g., diverse AI techniques, input features, and reported performance metrics such as AUC, sensitivity, specificity, or F1-score). No formal statistical quantification of heterogeneity (e.g., I^2^ statistic) was performed, as the studies were too clinically and methodologically diverse to support meaningful pooling of effect estimates in a meta-analysis—common in systematic reviews of AI-based prediction models where outcomes are heterogeneous and not uniformly reported as comparable effect sizes. These differences rendered quantitative synthesis infeasible and potentially misleading, leading to the decision to conduct a narrative synthesis instead.

This narrative synthesis directly aligns with the review’s objectives by prioritizing evidence according to clinical and predictive outcomes. Prediction-focused studies are grouped and discussed by key performance domains (e.g., discriminatory ability via AUC, sensitivity, specificity, accuracy, F1-score, calibration, and interpretability of risk factors such as knee valgus or landing mechanics), with comparisons across AI techniques (e.g., XGBoost and random forests vs. deep learning models) and validation methods (internal vs. external). Prevention-focused studies are synthesized by evidence of effectiveness, including reductions in ACL injury rates, biomechanical improvements, or outcomes of AI-driven interventions (e.g., real-time feedback or personalized programs), noting where prevention remains indirect via enhanced risk prediction. Cross-cutting factors such as specific AI approaches, sports contexts (e.g., soccer, female athletes), and limitations (e.g., small sample sizes, lack of prospective validation) are integrated within these outcome-based categories to explore sources of variation and facilitate interpretation. Performance metrics and key findings from all studies are summarized in Table 1, with the narrative highlighting patterns, strengths, evidence gaps, and implications for clinical translation in ACL injury prediction and prevention. This structured, outcome-oriented approach ensures the synthesis addresses the research questions on AI techniques, performance, contexts, and preventive evidence, supporting clear conclusions on best practices and future priorities.

## Results

**Fig. 1:**
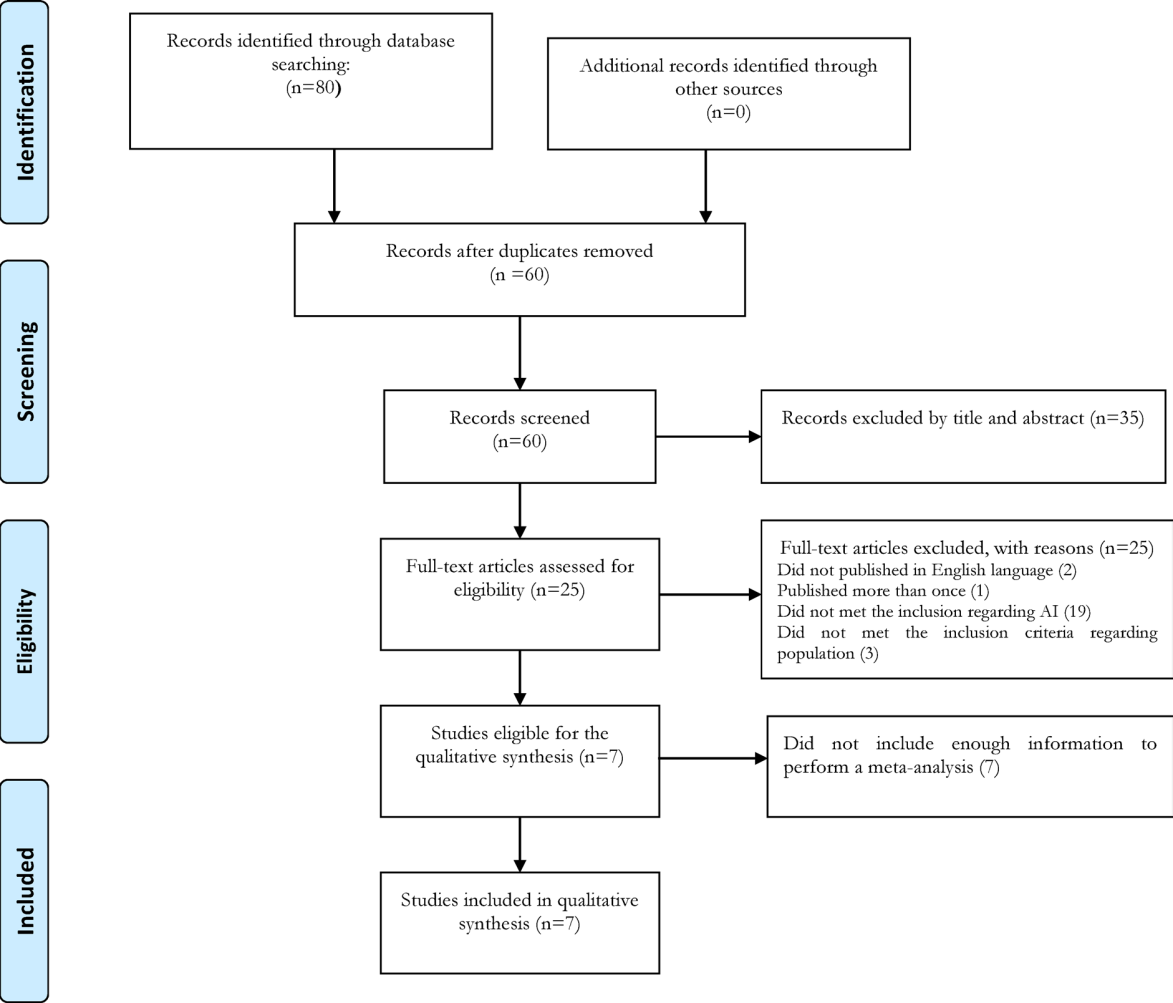
Flow diagram of systematic literature search. *AI* artificial intelligence

A total of 80 records were identified through database searches. After removing 20 duplicates, 60 records were screened by title and abstract, of which 35 were excluded for not meeting the inclusion criteria. The remaining 25 full-text articles were assessed for eligibility, and 18 were excluded due to non-English language, lack of focus on ACL or AI, or insufficient data. Seven studies were included in the final review. The study selection process is presented in the PRISMA flow diagram (Fig. 1).

### Study characteristics

The studies, published between 2019 and 2024, investigated the use of AI for predicting or preventing ACL injuries in various populations, including elite athletes (e.g., basketball, handball, soccer players) and healthy individuals, with sample sizes ranging from 5 to 880 participants and age ranges from 13 to 22.8 years. Input features varied widely, including biomechanical data (e.g., leg stability, joint kinematics, knee angles), motion capture data, and anthropometric or genetic data.

### Results of individual studies

All seven studies utilized AI techniques to predict or prevent ACL injuries, with applications focusing on either risk prediction or biomechanical assessment for prevention. Four studies [36–38, 40] primarily aimed to predict ACL injury risk, employing ML models such as Linear SVM (accuracy: 96%, F1-score: 95%) [36], Fine Gaussian SVM (accuracy: 79.5%, AUC: 0.85) [37], random forest regressor (MAE: 0.81°–8.73°) [38], and random forest with logistic regression (AUC-ROC: 0.63) [40]. Two studies [39, 42] focused on biomechanical assessments to inform prevention strategies, using Sparrow Search Algorithm-optimized ELM/LSTM (R2: 0.9947–0.9992) [39] and MediaPipe Pose (good concurrent validity with VICON) [42], respectively. One study [41] used a deep learning model (Convolutional Neural Network) to predict knee joint moments for risk assessment (correlation: 0.8895).

### Risk of bias in studies

The PROBAST + AI tool is a framework designed to assess the methodological quality of prediction model studies, specifically addressing concerns related to AI and ML. It extends the original PROBAST (Prediction Model Risk of Bias Assessment Tool) by incorporating AI-specific elements such as data preprocessing, model development, and generalizability. The tool evaluates ROB and applicability across four domains: Participants, Predictors, Outcome, and Analysis. Each domain is rated as low, high, or unclear for ROB and applicability concerns, providing a comprehensive assessment of the study's quality and relevance.

In Domain 1, Participants, all studies exhibited low ROB due to clear inclusion criteria and appropriate recruitment strategies, targeting athletes in relevant sports. Sample sizes varied significantly, ranging from n = 5 to n = 791, with larger cohorts, such as that of Jauhiainen et al. [40] enhancing the validity of the findings. Applicability was generally low for most studies, as the populations aligned with the review's focus on athletes; however, it was unclear for Xu et al. [39] (which included only males, despite a higher ACL risk in females) and Ohnishi et al. [38] (where a small sample size limited generalizability). In Domain 2, Predictors, all studies demonstrated low ROB, as predictors such as kinematics and sensor data were clearly defined, measured at baseline, and blinded where applicable. AI-specific aspects, including data preprocessing for machine learning inputs, were adequately described, leading to low applicability since the predictors were relevant to ACL biomechanics. Domain 3, Outcome, also showed low ROB, with outcomes like KAM, valgus angle, and ACL force determined using gold-standard methods (force plates and 3D motion capture) at consistent timing, with no noted outcome misclassification bias. The applicability remained low, as these outcomes were directly related to ACL risk prediction. In Domain 4, Analysis, a generally low ROB was observed, with appropriate handling of missing data, cross-validation to prevent overfitting, and the use of performance metrics such as area under the curve (AUC), mean absolute error (MAE), and R^2^. AI extensions in PROBAST + AI, such as hyperparameter optimization in Xu et al. [39] and permutation tests in Jauhiainen et al. [40], were adequately addressed, although the findings in Asaeda et al. [42] and Ohnishi et al. [38] were unclear due to small sample sizes that risked optimism bias. Applicability in this domain was low or unclear, as analyses primarily focused on lab data but were intended for field use. Overall, all studies demonstrated low ROB, indicating robust methodological quality for development. Applicability concerns were minimal, supporting their relevance to AI in ACL prevention and prediction; however, future studies should incorporate external validation and field testing to enhance generalizability (Table 2).

**Table 2:** PROBAST + AI ratings for included studies

References	Domain 1: participants (ROB/Applicability)	Domain 2: predictors (ROB/Applicability)	Domain 3: outcome (ROB/Applicability)	Domain 4: analysis (ROB/Applicability)	Overall, ROB	Overall applicability concerns
Benjaminse et al. [37]	Low/Low (n = 32 female athletes; lab-selected but target-relevant)	Low/Low (Kinematics from wearables; clear definition, no bias)	Low/Low (KAM from force plates; gold standard)	Low/Low (Cross-validation; AUC 0.81–0.85; no overfitting reported)	Low	Low
Asaeda et al. [42]	Low/Low (n = 15 participants; lab-based but focused on validity)	Low/Low (Pose estimation from video; reproducible)	Low/Low (Valgus angle from 3D motion; normalized)	Low/Unclear (Reliability tests; ICC high, but small n; no external validation)	Low	Low (Lab to field applicability unclear)
Jauhiainen et al. [40]	Low/Low (n = 880 female athletes; large cohort, real-world screening)	Low/Low (Biomechanical/3D motion data; well-defined)	Low/Low (ACL injury events; prospective)	Low/Low (Cross-validation + permutations; AUC 0.63; overfitting addressed)	Low	Low
Taborri et al. [36]	Low/Low (n = 39 female players; targeted population)	Low/Low (Inertial sensors/optics; clear protocols)	Low/Low (LESS score as risk proxy; validated)	Low/Low (Classifiers compared; accuracy 96%; cross-validation)	Low	Low
Johnson et al. [41]	Low/Low (Lab-simulated field; sports-relevant)	Low/Low (Motion data; CNN inputs)	Low/Low (KJM from force plates)	Low/Low (CNN with regression; correlation 0.89; validation splits)	Low	Low
Xu et al. [39]	Low/Low (n = 56 males; fatigue protocol)	Low/Low (Kinematics/joint angles; musculoskeletal inputs)	Low/Low (ACL force from simulation; validated)	Low/Low (SSA-optimized ELM/LSTM; R^2^ 0.99; cross-validation)	Low	Unclear (Male-only; applicability to females limited)
Ohnishi et al. [38]	Low/Low (n = 5; small but proof-of-concept)	Low/Low (Stretch sensor data; calibrated)	Low/Low (Valgus from 3D motion)	Low/Unclear (RFR; MAE 0.81°; small n risks overfitting)	Low	Unclear (Lab-based; field generalizability unclear)

### Results of syntheses

Due to heterogeneity in AI techniques, input features, study populations, and outcome measures, a narrative synthesis was conducted. The studies demonstrated a range of AI applications, with ML models (e.g., SVM, random forest) and deep learning (e.g., CNN) showing promise in predicting ACL injury risk and assessing biomechanical factors for prevention. Performance metrics varied, with accuracy ranging from 79.5 to 96% and AUC from 0.63 to 0.85 in risk prediction studies, while prevention-focused studies reported high predictive accuracy (e.g., R2: 0.9947–0.9992) or good validity. The heterogeneity was attributed to differences in AI methodologies, input data (e.g., biomechanical, kinematic, or video-based), and study populations (e.g., elite athletes vs. healthy individuals).

## Discussion

This systematic review investigates the role of AI in predicting and preventing ACL injuries by synthesizing findings from seven studies. The following sections critically analyze the functional tests employed, input data utilized, ML methods applied, clinical applications, participant characteristics, study quality, and limitations, contextualizing these findings within the broader literature to highlight implications for clinical practice and future research.

### Functional tests and AI predictive capacity

The studies employed diverse functional tests to assess ACL injury risk, with predictive accuracy linked to the relevance of tasks to injury mechanisms. Taborri et al. [36] achieved 96% accuracy in identifying high-risk athletes using single-leg squats and jumps, likely due to their ability to detect neuromuscular control deficits, a known contributor to ACL injuries [44]. In contrast, Jauhiainen, Kauppi [40] reported a modest AUC-ROC of 0.63 with vertical drop jumps (VDJ) and cutting maneuvers, suggesting that VDJ may not fully replicate injury scenarios such as pivoting, as noted in their discussion. Xu et al. [39] and Asaeda et al. [42] focused on single-leg landings, with Xu’s model excelling in force prediction (high R^2^) through ankle kinematics, while Asaeda’s 2D MediaPipe Pose showed moderate validity (r = 0.554–0.757) due to dimensional constraints. Benjaminse et al. [37] utilized agility tasks, achieving 79.5% accuracy in classifying knee abduction moments (KAM), which aligns with the demands of dynamic sports like football. Johnson et al. [41] assessed walking, running, and sidestepping, reporting a high correlation (r = 0.9277) for knee joint moments (KJM), demonstrating AI’s capability in modeling complex movements. Ohnishi et al. [38] evaluated 13 diverse movements, achieving an MAE of 0.81° for valgus angle with user specific data, highlighting the versatility of multi-task assessments. These findings suggest that AI can enhance early risk detection, particularly when tests mimic injury mechanisms, but practical challenges, such as the need for specialized equipment, must be addressed for broader adoption.

### Input data

Input data varied across studies, influencing both predictive accuracy and clinical feasibility. Taborri et al. [36] used biomechanical parameters (e.g., Ellipse Area, RMS) from inertial and optoelectronic sensors, offering high precision but requiring laboratory settings. Xu et al. [39] focused on ankle kinematics (e.g., Ankle Contact Index), effectively capturing dynamic patterns, while Benjaminse et al. [37] and Johnson et al. [41] employed full-body kinematics via IMUs and Vicon systems, providing comprehensive insights at the expense of portability. Jauhiainen et al. [40] incorporated 283 variables, which may have contributed to overfitting due to data complexity. Asaeda et al. [42] relied on 2D video analysis, enhancing accessibility but introducing errors in absolute DKV (18.83°–19.68°). Ohnishi et al. [38] utilized stretch sensors for real-time monitoring, improving field applicability despite calibration requirements. This balance between precision and practicality indicates that AI tools can be adapted for diverse clinical settings, but ethical concerns, such as data privacy in real-time monitoring, must be addressed to ensure responsible use [45]. Data selection should align with the intended clinical purpose, such as screening or on-field monitoring.

### Machine learning methods

The choice of ML algorithms influenced study outcomes. Taborri et al. [36], Jauhiainen et al. [40], Benjaminse et al. [37] applied support vector machine (SVM) variants, suitable for biomechanical classification due to their ability to handle highdimensional data, though Jauhiainen’s lower AUC-ROC (0.63) suggests potential overfitting with 283 variables. Johnson et al. [41] used a convolutional neural network (CNN), achieving r = 0.9277 for KJM prediction, leveraging deep learning’s strength in pattern recognition, albeit with increased computational demands. Xu et al. [39] combined SSA, ELM, and LSTM, optimizing force prediction for dynamic tasks. Asaeda et al. [42] employed MediaPipe Pose, but 2D limitations reduced DKV accuracy (r = 0.006–0.590). Ohnishi et al. [38] used random forest regressor (RFR), outperforming Linear SVR (MAE = 0.81° vs. 1.33° for valgus), likely due to its robustness to sensor noise. These differences highlight the need to tailor algorithms to data types and task complexity; SVMs are effective for straightforward classification, while neural networks excel in dynamic predictions. However, the opacity of some models (e.g., neural networks) raises ethical concerns about transparency in clinical decision-making. Algorithm selection should balance accuracy with interpretability for practical use.

### Clinical applications

AI tools demonstrated varied clinical applications for ACL injury prevention. Taborri et al. [36] proposed operator-independent screening, ideal for large-scale risk assessments in sports teams, potentially enabling early interventions. Benjaminse et al. [37] and Ohnishi et al. [38] utilized wearable sensors (IMUs and stretch sensors), with Ohnishi’s real-time alerts supporting on-field adjustments, though calibration remains a practical challenge. Asaeda et al. [42] introduced 2D video analysis, a cost-effective option for multicenter studies, despite its lower precision. Xu et al. [39] emphasized fatigue monitoring, critical for high-intensity sports, while Johnson et al. [41] indirectly supported load management through KJM prediction. Jauhiainen et al. [40] showed potential for understanding injury causation, though limited by variable selection. These tools can facilitate personalized training programs, but high development costs and ethical concerns regarding data privacy may hinder widespread adoption [45]. Collaboration with clinicians is essential to overcome these barriers and integrate AI into routine practice.

### Participant characteristics

Participant demographics varied, affecting the generalizability of findings. Taborri et al. [36] studied young female basketball players, reflecting the higher ACL injury risk in females due to biomechanical differences [46]. Jauhiainen et al. [40] and Benjaminse et al. [37] focused on elite female handball/soccer players and young female footballers, respectively, further emphasizing gender-specific risks. Xu et al. [39] and Asaeda et al. [42] included healthy males, potentially overlooking female-specific factors. Johnson et al. [41] provided limited demographic details, restricting interpretability. Ohnishi et al. [38] involved a mixed-gender cohort (3 males, 2 females, mean age 22.8 years) with a small sample size (n = 5). These demographic differences suggest that AI models may require gender-specific tuning, a practical consideration for future validation studies, with ethical implications for ensuring equitable data representation [45].

### Study quality assessment

The ROB and applicability assessments across seven recent studies (2019–2024) employing ML or advanced modeling to predict ACL injury risk or related biomechanical proxies (e.g., knee abduction moment [KAM], valgus angle, or joint loading) in athletic populations, predominantly females at elevated risk, indicate a consistent low risk of bias. Overall, the evaluations indicate consistently low ROB across all domains participant selection, predictors (wearables, video pose estimation, inertial sensors, or 3D motion capture), outcomes (gold-standard force-plate measures or prospective injury events), and analysis (cross-validation, permutation tests, or high-performance metrics like AUC 0.63–0.85, accuracy 96%, or R^2^ 0.99) reflecting methodologically robust designs with appropriate validation strategies to mitigate overfitting and ensure reproducibility. Applicability concerns remain low in most cases due to targeted female athlete cohorts, sport-relevant tasks, and field-applicable technologies (e.g., wearables or video-based systems), though limitations emerge in smaller-sample proof-of-concept works (e.g., n = 5 or n = 15) and male-only or lab-confined protocols, which introduce uncertainty regarding generalizability to female athletes or real-world field settings. These findings highlight substantial progress in developing low-bias, clinically promising predictive models for ACL injury prevention, yet underscore the need for larger prospective validations, female-inclusive cohorts, and external field testing to bridge remaining gaps toward practical screening implementation.

### Limitations and future scope

Common limitations across studies include small or homogeneous samples, restricting generalizability. The focus on specific movements may miss broader injury mechanisms. Laboratory-based data e.g., Vicon and 2D constraints reduce ecological validity, limiting real-world applicability. Calibration requirements and variable overload present practical challenges. The heterogeneity of study designs precluded meta-analysis. Ethical concerns, such as data privacy in real-time monitoring, and practical barriers, including high costs, further complicate adoption. Future research should prioritize larger, more diverse cohorts, multi-modal data integration, and standardized protocols to enhance clinical applicability.

## Conclusion

AI demonstrates significant potential in predicting ACL injury risk and informing prevention strategies through biomechanical and kinematic analyses. However, small sample sizes, heterogeneous methodologies, and practical barriers (e.g., equipment costs) limit clinical adoption. Future research should focus on larger, diverse cohorts and standardized protocols to enhance generalizability and implementation.

## Supplementary Information

Below is the link to the electronic supplementary material.

Supplementary Material 1

Supplementary Material 2

## Data Availability

The datasets used and/or analyzed during the current study are available from the corresponding author on reasonable request.
